# ﻿Revision of the Chinese *Pachynotus* Kollar & L. Redtenbacher, 1844 (Coleoptera, Curculionidae), with descriptions of two new species

**DOI:** 10.3897/zookeys.1197.114969

**Published:** 2024-04-10

**Authors:** Jinliang Ren, Li Ren, Runzhi Zhang

**Affiliations:** 1 Key Laboratory of Zoological Systematics and Evolution, Institute of Zoology, Chinese Academy of Sciences, No.1 Beichen West Road, Chaoyang District, Beijing 100101, China Institute of Zoology, Chinese Academy of Sciences Beijing China; 2 College of Life Science, University of Chinese Academy of Sciences, Beijing 100049, China University of Chinese Academy of Sciences Beijing China

**Keywords:** COI, Curculionidae, key, new species, *Pachynotus*, Xizang

## Abstract

The Chinese species of the highland weevil genus *Pachynotus* is revised, including a single known species, *P.lampoglobus* Chao & Y.-Q. Chen, 1980, and the descriptions of two new species, *P.pilosus***sp. nov.** and *P.arcuatus***sp. nov.** All Chinese *Pachynotus* species occur in Xizang (Tibet), China, and a key to these species is presented. Additionally, the COI sequences of two species, *P.lampoglobus* and *P.pilosus***sp. nov**., are provided, with details of the genetic distance.

## ﻿Introduction

*Pachynotus* Kollar & L. Redtenbacher, 1844, belonging to the tribe Tanymecini (Curculionidae, Entiminae), is mainly distributed in the Himalayas, such as Kashmir, Himachal Pradesh, Uttarakhand of India and Xizang of China (Alonso-Zarazaga et al. 2023; Appendix 1). Because of the Himalayas’ high species richness and endemism, this mountain region has been identified as one of the 25 biodiversity hotspots in the world (Myers et al. 2000). The genus *Pachynotus* was established by Kollar and Redtenbacher (1844) for a single species, *P.globicollis* Kollar & L. Redtenbacher 1844, from India and Kashmir. Marshall (1916) redefined the genus and described *P.globicollis*, while synonymising *Cneorhinusobscurus* Kollar & L. Redtenbacher, 1844 with *P.globicollis*. Chao and Chen (1980) described *P.lampoglobus* Chao & Y.-Q. Chen, 1980 from China. Mahendiran and Ramamurthy (2013) reviewed this genus, described two new species, *P.mayarami* Mahendiran & Ramamurthy, 2013 and *P.kumaonensis* Mahendiran & Ramamurthy, 2013, and provided a key to all the species distributed in India.

Herein, we describe two new species and present a key to Chinese *Pachynotus*. Moreover, we provide the COI sequences of *P.lampoglobus* and *P.pilosus* and analyse the genetic distance of these two species based on COI sequences.

## ﻿Materials and methods

All specimens, including types, examined for this study are collections of the Institute of Zoology, Chinese Academy of Sciences, Beijing, China (**IZCAS**), and the Natural History Museum, London, UK (**NHMUK**). The types of the new species are deposited in IZCAS. Label data are given as they are, verbatim, with pinyin romanisation and comments in square brackets if labels are in Chinese; labels are separated by double slashes and lines by slashes.

Specimens were dissected after soaking them in soapy water overnight for cleaning and softening, and the dissected parts were placed in a cold 10% KOH solution for 20 h to macerate the soft tissues. After dissection, all parts were photographed and stored in glycerine in microvials pinned beneath the specimen from which they were dissected.

The morphological terminology used in this study mainly follows Marshall (1916) and Aslam (1966). Measurements were made using an ocular micrometre as follows: standard length – dorsally from anterior margin of thorax to the apex of elytra along midline; pronotal length – dorsally from anterior margin to base along midline; pronotal width – dorsally across widest part; elytral length – dorsally along suture of elytra from base to apex; elytral width – dorsally across widest part; rostral length – dorsally in a straight line from apex to anterior margin of eye; rostral width – dorsally across base of rostrum. Measurements are made in millimetres.

All observations and dissections were performed using a Nikon SMZ1500 stereomicroscope. The habitus images were taken with a Canon-5D camera mounted on a Nikon SMZ1500 microscope. CombineZM and Helicon Focus software were used to combine the photos. Photoshop CC2019 was used to modify the photos if required.

DNA was extracted from all the specimens via DNeasy Blood & Tissue Kits (Qiagen, Germany). DNA was extracted from either 1, 3, or 6 legs or the whole body, depending on the size of specimen. Polymerase chain reaction (PCR) amplifications for COI sequences were conducted using the primers LCO1490 (GGTCAACAAATCATAAAGATATTGG) and HCO2198 (TAAACTTCAGGGTGACCAAAAAATCA). PCR reaction mixes (25 mL) contained 12.5 μL 2× Taq PCR MasterMix (Tiangen Biotech Co., Ltd, Beijing, China), 1 μL of forward and reverse primer each (Sangon Biotech Co. Ltd, Shanghai, China), 2 μL total undiluted DNA template, and 8.5 μL dd H_2_O. PCR profile as follows: 94 °C for 2 min, first cycle set (5 repeats): 94 °C for 40 s, 45 °C for 40 s and 72 °C for 60 s. Second cycle set (35 repeats): 94 °C for 40 s, 51 °C for 40 s and 72 °C for 60 s, followed by elongation at 75 °C for 5 min. PCR products were visualised through 1% agarose gel electrophoresis in TAE buffer. Successful PCR products were sent for sequencing in the Beijing Genomics Institute (BGI, Shenzhen, China). The raw data were assembled and edited via SeqMan v. 7.1. We failed to amplify the COI sequence of *P.arcuatus*. In order not to destroy the type specimen (only one specimen), we abandoned the idea of further amplification of the COI sequence. The intraspecific and interspecific K2P genetic distances of *P.lampoglobus* and *P.pilosus* were separately calculated using the MEGA v. 7.

## ﻿Results

### ﻿Taxonomic treatment

#### ﻿ Pachynotus

Taxon classificationAnimaliaColeopteraCurculionidae

Kollar & L. Redtenbacher, 1844

A336D6F9-176B-53F5-A005-DAA0A194B2EC

Pachynotus Kollar & L. Redtenbacher 1844: 541. Type species: P.globicollis Kollar & L. Redtenbacher, 1844. Type locality: India and Kashmir.

##### Diagnosis.

Head with eyes lateral, ovate, flat, or slightly prominent. Base of rostrum not or only slightly broader than forehead, with narrow or broad central furrow, reaching fore margin of forehead or head apex; antennal scrobes dilated and much shallower behind, upper edge touching eyes. Antennal with scape reaching the middle or posterior margin of the eyes at rest, clavate; funicle with the two basal desmomeres more elongate, desmomere III–VI subequal, desmomere VII subconical; club short, ovate, three-segmented. Pronotum with a central sulcus, fine, extremely shallow. Elytra ovate, with base raised as prominent flange or not, and the base bisinuate or not; odd interstriae more raised than even ones or not; metanepisternum fused with it behind, metanepisternal suture therefore distinct only in the cephalic 1/2. Corbels of hind tibiae open or narrowly enclosed; claws connate at base.

#### ﻿ Pachynotus pilosus sp. nov.

Taxon classificationAnimaliaColeopteraCurculionidae

A9AD1D38-6DE0-55E3-9A76-9FD6D4A5BFA8

https://zoobank.org/37B015C6-3A67-4BD0-B8D7-F312C395791F

**Figure 1.**
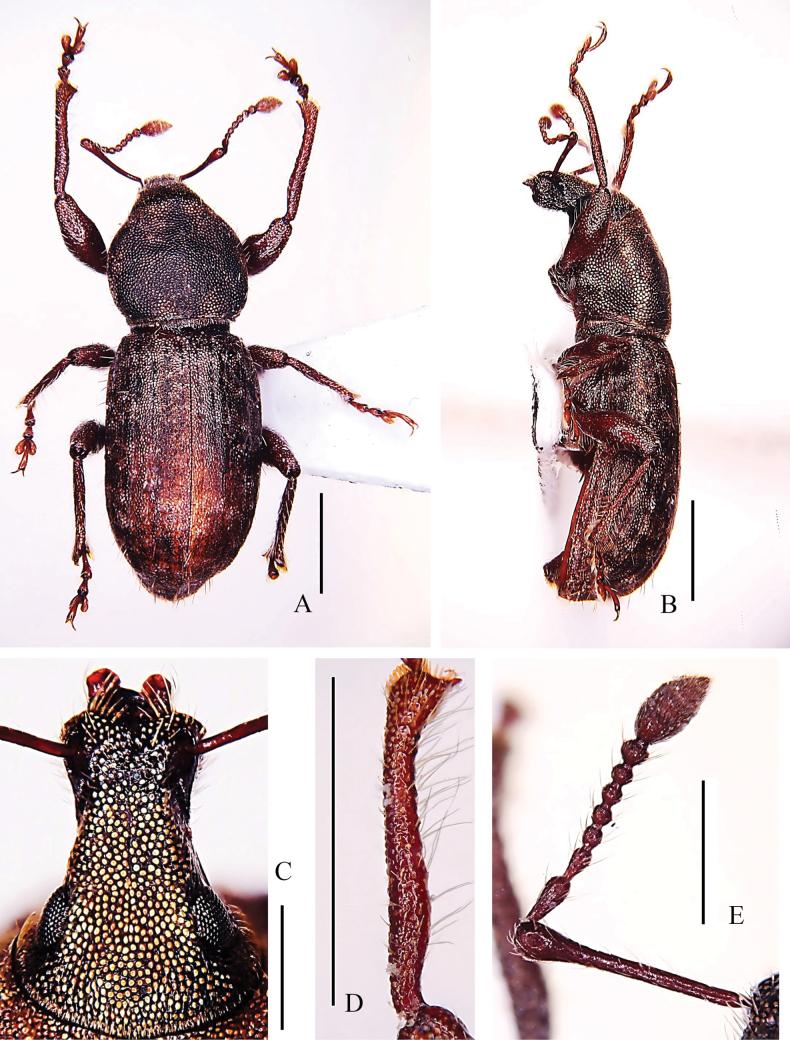
Habitus of *Pachynotuspilosus* sp. nov., holotype **A** dorsal view **B** lateral view **C** head and rostrum, dorsal view **D** left protibia, dorsal view **E** antenna, dorsal view. Scale bars: 1 mm (**A, B, D**); 0.5 mm (**C, E**).

**Figure 2.**
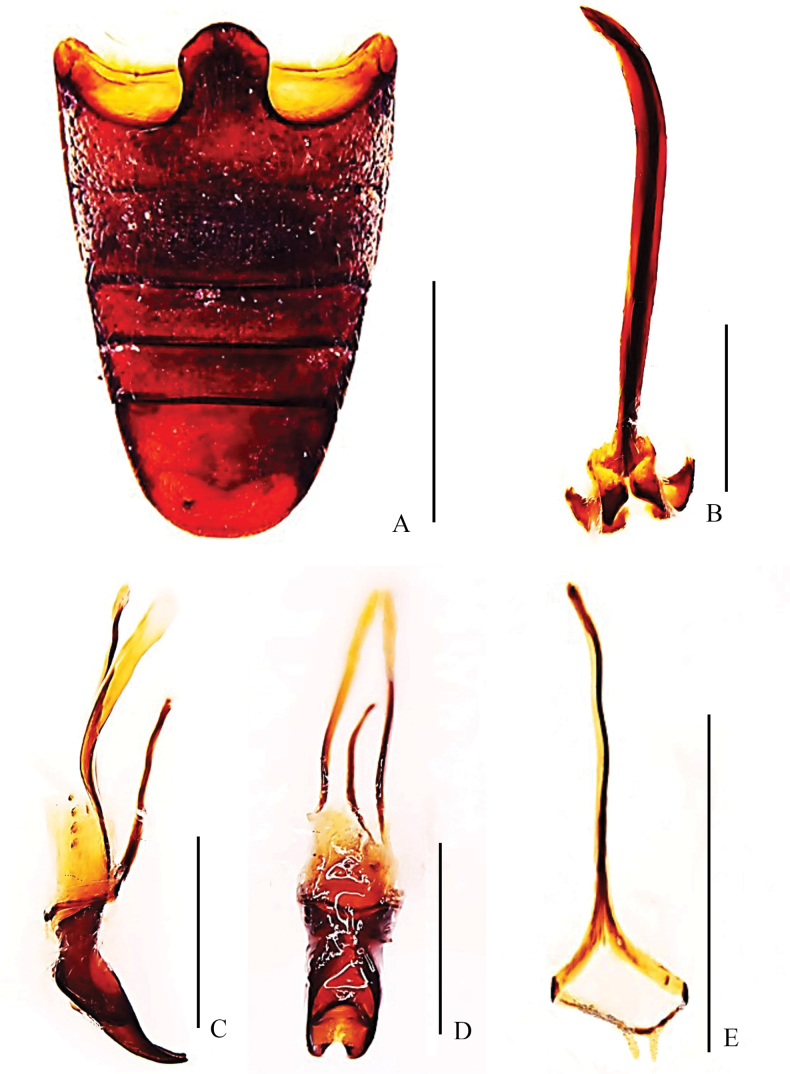
Abdominal and genital structures of *Pachynotuspilosus* sp. nov., holotype **A** ventrites, ventral view **B** sternites VIII and IX, dorsal view **C** aedeagus, lateral view **D** aedeagus, dorsal view **E** tegmen, dorsal view. Scale bars: 0.5 mm.

**Figure 3.**
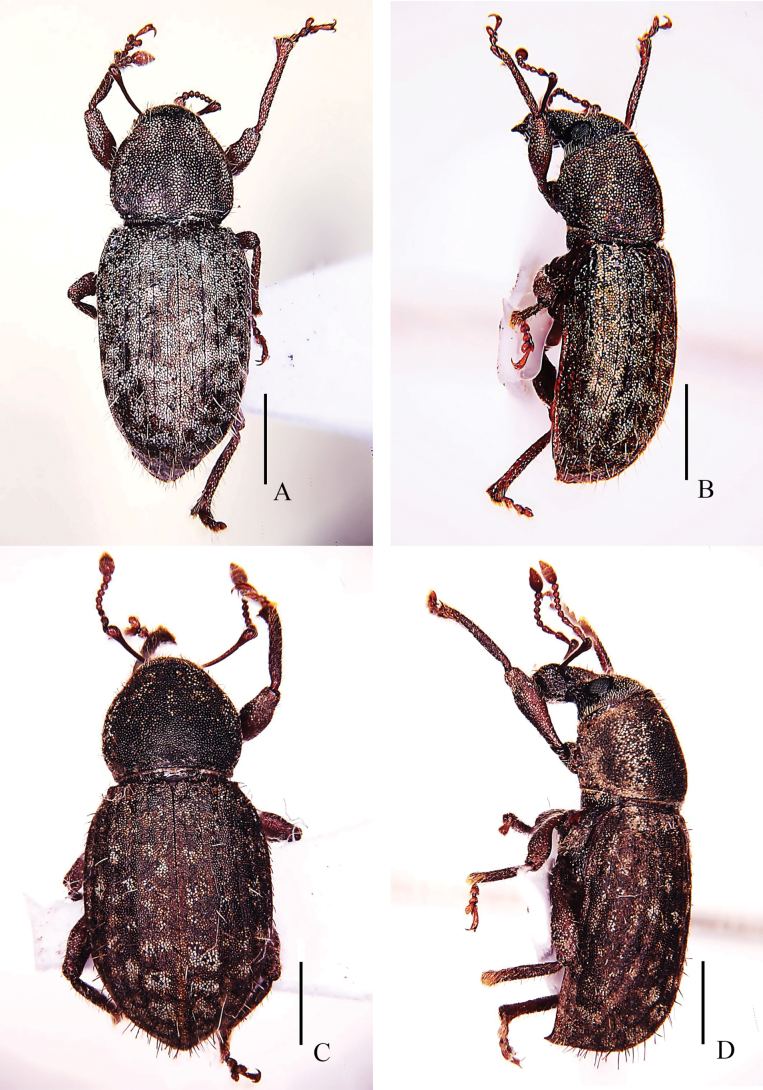
Habitus of *Pachynotuspilosus* sp. nov. paratype **A** dorsal view **B** lateral view. *Pachynotusarcuatus* sp. nov. holotype **C** dorsal view **D** lateral view. Scale bars: 1 mm.

**Figure 4.**
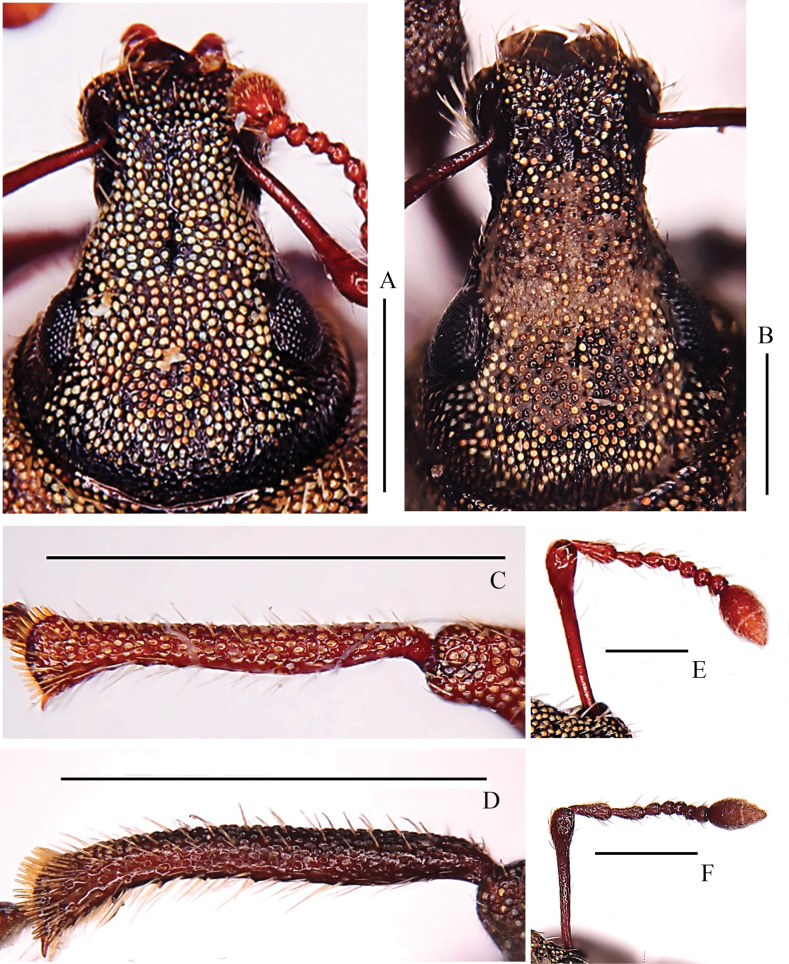
*Pachynotuspilosus* sp. nov. **A** head and rostrum, dorsal view **C** right protibia, anterior view **E** antenna, anterior view; *Pachynotusarcuatus* sp. nov. **B** head and rostrum, dorsal view **D** right protibia, anterior view **F** antenna, anterior view. Scale bars: 0.5 mm (**A, B, E, F**); 1 mm (**C, D**).

**Figure 5.**
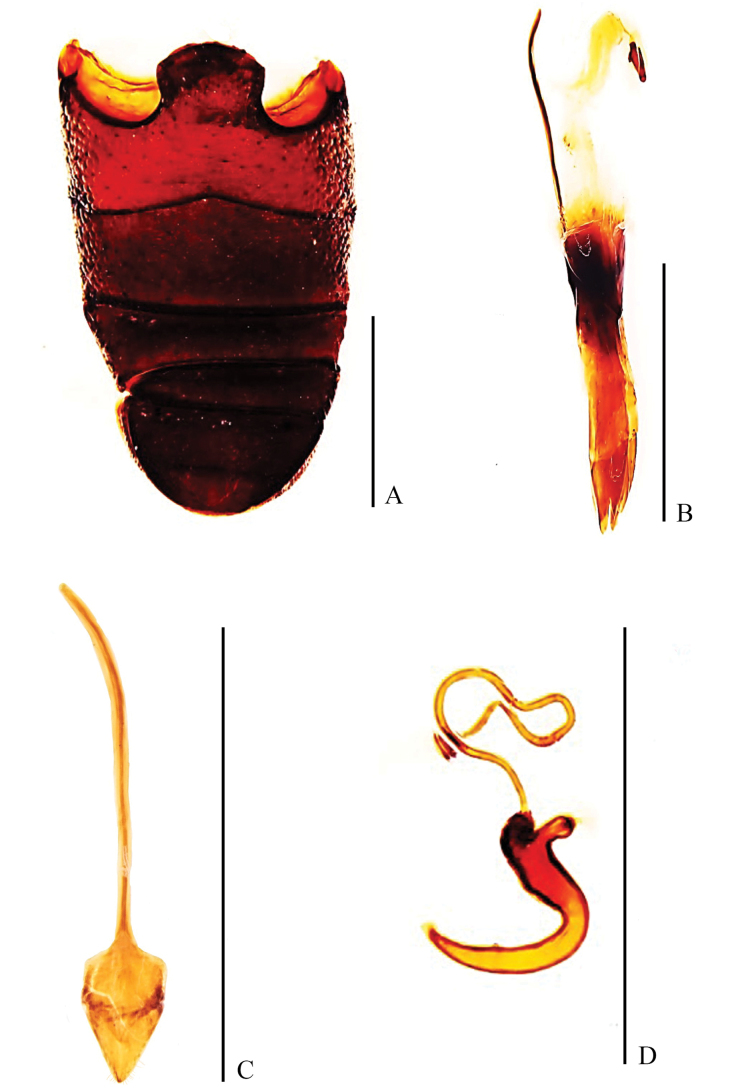
Abdominal and genital structures of *Pachynotuspilosus* sp. nov. **A** ventrites, ventral view **B** sternite VIII and female genitalia, lateral view **C** sternum VIII, dorsal view **D** spermatheca, lateral view. Scale bars: 0.5 mm.

Figs 1 , 2 , 3A, B , 4A, C, E , 5

##### Material examined.

***Holotype***, ♂: (white, printed): 西藏山南曲松 [Xīzàng Shānnán Qǔsōng] 邱多江乡亚堆扎拉山垭口 [Qiūduōjīangxīang Yàduīzhālāshān yàkǒu] / 28.8307°N, 92.0507°E / 中科院动物所 [Zhōngkēyuàn Dòngwùsuǒ] / 4989.19 m / 2021.VII.12 / 任金梁 [Rén Jīnliáng]: IOZ(E)1965015 // ***Paratypes*** (2♂, 3♀): same data as holotype except IOZ(E)1965016–IOZ(E)1965020.

##### Description.

**Holotype, male.** Measurements (in mm): standard length: 5.10; pronotal length: 1.70; pronotal width: 1.9; elytral length: 3.10; elytral width: 2.08; rostral length: 0.90; rostral width: 0.60.

***Habitus and colour*** (Fig. 1A, B): body elongate-oval, small; integument dark reddish brown; antennae and legs reddish brown, with pale yellowish-brown scales; scales on dorsal and lateral surfaces of rostrum moderately dense, oval to elongate-oval; antennal scape and funicles without scales; pronotum with polygonal scales, moderately dense, not contiguous; scales on elytra polygonal, dense, not contiguous; scales on ventrites moderately dense, polygonal, elongate-oval; scales on legs dense; dorsal of tarsi surface without scales; body with erect to suberect and slightly longer setae; rostrum sparsely covered with suberect fine setae; antennal scape and desmomeres I–VII with long, fine, sparse setae; dorsal and lateral surfaces of pronotum with sparse, suberect setae; setae on interstriae long, erect to suberect, behind declivity slightly longer, erect, equal to 6 × diameter of one scale; setae on ventral surface moderately long, fine, erect.

***Head*** (Fig. 1C): moderately convex; dorsal surface smooth; punctures small, moderately dense; eyes flat, oval; forehead weakly convex, moderately elevated than base of rostrum in lateral view.

***Rostrum*** (Fig. 1C): in dorsal view 1.50 × as long as wide, apex narrower than base; sides narrowed from base to middle, and then slightly broadened to apex; dorsal surface with a narrow and shallow median sulcus, reaching fore margin of forehead; epistome broad U-shaped, smooth, posterior angle of epistome >90°; mandibular scars oval; ventral margin of antennal scrobes visible at apical half in dorsal view; in lateral view, without triangular depression positioned laterally between eyes and antennal scrobes; prementum with two setae.

***Antennae*** (Fig. 1E): scape slender, clavate, reaching posterior margin of eyes at rest, 0.99 × length of funicle; desmomere I 1.40 × length of II, both segments elongate clavate; desmomere III short, clavate, 0.59 × length of II; desmomere IV 0.91 × length of III, nearly equal width; desmomeres IV–VI moniliform, equal in length and width; desmomere VII moniliform, 1.21 × length, 1.24 × width of VI; club elongate-oval, apical sharp, three-segmented, uniformly pubescent, segment I 1.25 × length of II; segment II shorter than segment III.

***Pronotum***: 0.89 × as long as wide; subquadrate in dorsal outline, strongly convex; sides strongly rounded, greatest width after midpoint, gradually narrowing towards both ends, fore margin shorter than posterior one; median sulcus fine, extremely shallow; dorsal surface smooth, punctures small, each puncture covered by a scale; postocular lobes absent, vibrissae fine, dense, yellow.

***Scutellum***: large and distinct, triangular, shiny, uncoated, reddish brown.

***Elytra***: 1.49 × as long as wide, moderately convex, elongate-oval; base not raised as prominent flange, not bisinuate; sides subparallel before declivity, only slightly narrowed near the base; striae distinct, narrow, moderately deep, linear; punctures minute, spot like, moderately dense, space between punctures narrower than the diameter of punctures; interstriae wide, slightly flat, without tubercles, odd interstriae slightly raised than even ones.

***Abdomen*** (Fig. 2A): sternite I depressed in middle, slightly convex at sides; suture between sternites I and II slightly curved forward in middle; sternite II slightly convex; sternite I longer than II, sternite II slightly shorter than III and IV combined; sternites III and IV equal in length; sternite V moderately convex, apex round, longer than sternites III and IV combined.

***Legs***: rather short; femora clavate; fore tibiae subcylindrical, slightly sinuate, slightly bent inwards at apical quarter, apex not projected inwards and outwards, with long and slender hairs, obviously different from other setae, somewhat sparse (Fig. 1D); inner margin of fore tibiae with several extremely small teeth; median and hind tibiae without teeth; corbels of hind tibiae closed.

***Genitalia***: sternite VIII (Fig. 2B) divided into two hemisternites, transversely oriented, subtrapeziform; each laterally acuminate. Sternite IX (Fig. 2B) with basal plate bilobed; spiculum gastrale 1.19 × as long as aedeagus, strongly sclerotised, anterior third curved. Penis (Fig. 2C, D) in dorsal view with tube 0.97 mm in length, 4.8 × length of wide, temones 0.62 mm long; in lateral view curved, more strongly so near base and at apex, greatest width at midpoint. Tegmen (Fig. 2E) 0.61 × length of penis, with ring narrow, parameroid lobes developed with basal half more sclerotised; tegminal apodeme slender, forming a Y-shape with basal piece.

##### Variation.

**Male paratypes.** Measurements (in mm): standard length: 5.00–5.30; pronotal length: 1.7–1.9; pronotal width: 1.85–2.10; elytral length: 3.00–3.20; elytral width: 2.00–2.20; rostral length: 0.80–1.00; rostral width: 0.60–0.70.

**Females.** Measurements (in mm): standard length: 4.80–5.50; pronotal length: 1.70–1.80; pronotal width: 1.80–2.10; elytral length: 3.10–3.50; elytral width: 2.08–2.60; rostral length: 0.80–1.00; rostral width: 0.60–0.75. Fore tibiae with short and slender setae, same as other setae (Fig. 4C); ventrite V (Fig. 5A) parabolic, longer than ventrite II. Sternite VIII (Fig. 5C) with spiculum ventrale thin, rod-like and sinuate. Spermatheca (Fig. 5D) with corpus subquadrate; cornu widely curved, U-shaped, apically gradually narrowed; ramus subspherical, prominent and long.

##### Etymology.

*Pilosus*, Latin adjective, meaning “hairy”, in reference to the significantly longer hair on the male fore tibiae than on other parts.

##### Distribution.

China (Xizang).

#### ﻿ Pachynotus arcuatus sp. nov.

Taxon classificationAnimaliaColeopteraCurculionidae

AE60747A-CFC6-5B30-B97C-2ABFF56082D8

https://zoobank.org/1D3535AB-C051-4DBE-9458-390738E4B13E

**Figure 6.**
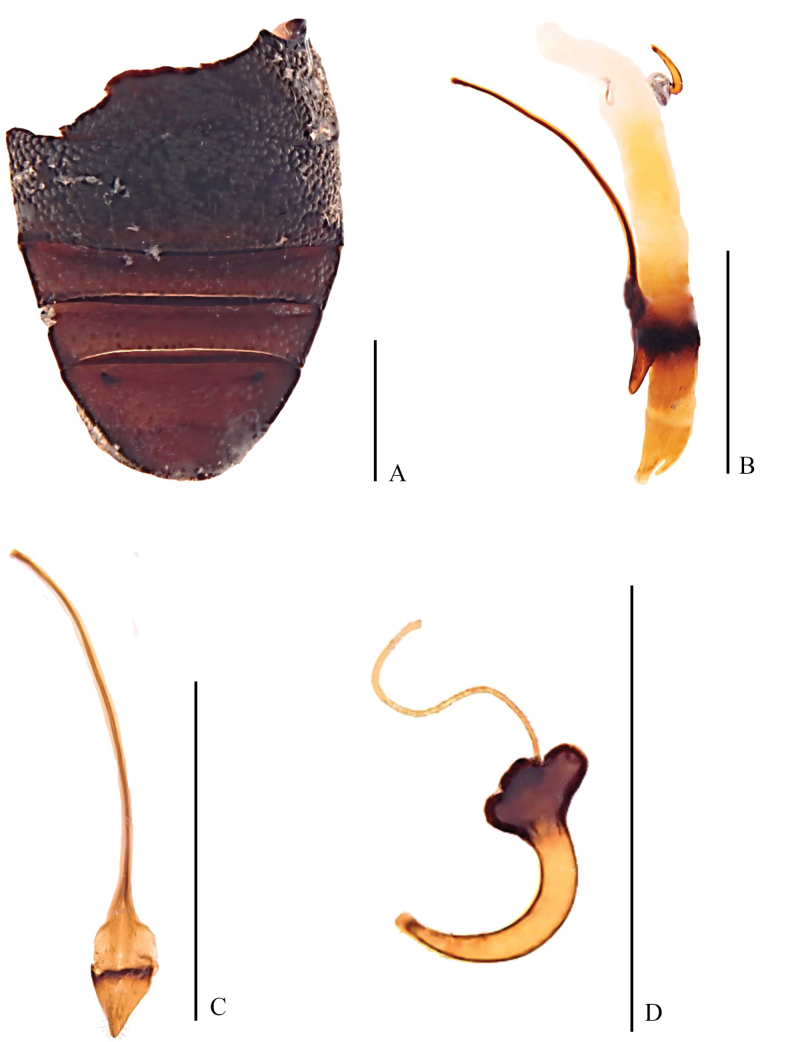
Abdominal and genital structures of *Pachynotusarcuatus* sp. nov. **A** ventrites, ventral view **B** sternite VIII and female genitalia, lateral view **C** sternum VIII, dorsal view **D** spermatheca, lateral view. Scale bars: 0.5 mm.

Figs 3C, D , 4B, D, F , 6

##### Material examined.

***Holotype***, ♀: (white, printed): 西藏山南地区 [Xīzàng Shānnándìqǖ] 羊措拉山 [Yángcuòlāshān] / 28.09708°N, 91.933395°E / 4685 m / 2018.VIII.15 / 周润 [Zhōu Rùn] / 中科院动物所 [Zhōngkēyuàn Dòngwùsuǒ]: IOZ(E)1965679.

##### Description.

**Holotype, female.** Measurements (in mm): standard length: 5.90; pronotal length: 1.90; pronotal width: 2.20; elytral length: 3.90; elytral width: 3.00; rostral length: 1.15; rostral width: 0.85.

***Habitus and colour*** (Fig. 3C, D): body elongate-oval, small; integument dark reddish brown; antennae and legs reddish brown, with light-grey scales; scales on dorsal and lateral surfaces of rostrum moderately dense, oval to elongate-oval; antennal scape and funicles without scales; pronotum with polygonal scales, moderately dense, not contiguous; scales on elytra polygonal, dense, not contiguous; scales on ventrites moderately dense, polygonal to elongate-oval; scales on legs dense; dorsal of tarsi surface without scales; body with erect to suberect and slightly longer setae, sparser; rostrum sparsely covered with suberect fine setae; antennal scape and desmomeres I–VII with long, fine and sparse setae; dorsal and lateral surfaces of pronotum with sparse, suberect setae; setae on interstriae long, erect to suberect, behind declivity slightly longer, erect, equal to 6 × diameter of one scale; setae on ventral surface moderately long, fine, erect.

***Head*** (Fig. 4B): moderately flat; dorsal surface smooth; punctures small and dense; eyes flat, oval; forehead weakly convex, moderately elevated than base of rostrum in lateral view.

***Rostrum*** (Fig. 4B): in dorsal view, 1.35 × as long as wide, apex narrower than base; sides narrowed from base to middle, and then slightly broadened to apex; dorsal surface with a broad, deep, median sulcus, reaching vertex of head; posterior angle of epistome 90°, smooth; mandibular scars oval; ventral margin of antennal scrobes visible at apical half in dorsal view; in lateral view, without triangular depression positioned laterally between eyes and antennal scrobes; prementum with two setae.

***Antennae*** (Fig. 4F): scape slender, clavate, reaching posterior margin of eyes at rest, 0.98 × length of funicle; desmomere I 1.43 × length of II, both segments elongate clavate; desmomere III short, clavate, 0.51 × length of II; desmomere IV 0.93 × length of III, nearly equal width; desmomeres IV–VI moniliform, equal in length and width; desmomere VII moniliform, 1.08 × length, 1.03 × width of VI; club elongate-oval, apically sharp, three-segmented, uniformly pubescent, segment I 1.21 × length of II; segment II shorter than segment III.

***Pronotum***: 0.86 × as long as wide; subquadrate in dorsal outline, strongly convex; sides strongly rounded, greatest width after midpoint, gradually narrowing towards both ends, fore margin shorter than posterior one; median sulcus fine, extremely shallow; postocular lobes absent, vibrissae fine, dense, yellow.

***Scutellum***: large and distinct, U-shaped, shiny, uncoated, reddish brown.

***Elytra***: 1.30 × as long as wide, moderately convex, elongate-oval; with base raised as prominent flange, not bisinuate; sides subparallel before declivity, only slightly narrowed near base; striae distinct, narrow, moderately deep, linear; puncture minute, spot-like, moderately dense, space between punctures narrower than diameter of punctures; interstriae wide, slightly flat, without tubercles, odd interstriae slightly raised than even ones.

***Abdomen*** (Fig. 6A): sternite I depressed in middle, slightly convex at sides; suture between sternites I and II slightly bisinuate; sternite II slightly convex; sternite I longer than II, sternite II slightly shorter than III and IV combined; sternites III and IV equal in length; sternite V moderately convex, apical round, longer than sternites III and IV combined.

***Legs***: rather short; femora clavate; fore tibiae obviously bent inward at apical third, apex neither projected inwards nor outwards (Fig. 4D); inner margin of fore tibiae with several extremely small teeth; median- and hind tibiae without teeth; corbels of hind tibiae closed.

***Genitalia***: ventrite V (Fig. 6A) parabolic, longer than ventrite II. Sternite VIII (Fig. 6C) with spiculum ventrale thin, rod-like, and sinuate. Spermatheca (Fig. 6D) with corpus subquadrate; cornu elongate, widely curved, wide U-shaped, apically gradually narrowed; ramus quadrate.

**Male.** Unknown.

##### Remarks.

This new species resembles *P.pilosus* sp. nov. but differs by the following characters: scutellum U-shaped; rostrum with a broad and deep median sulcus, reaching head vertex; elytra base raised as prominent flange; fore tibiae obviously bent inward at apical third.

##### Etymology.

*Arcuatus*, Latin participle, meaning “curved”, in reference to the fore tibiae, which is bent inward at its apical third.

##### Distribution.

China (Xizang).

#### ﻿ Pachynotus lampoglobus

Taxon classificationAnimaliaColeopteraCurculionidae

Chao & Y.-Q. Chen, 1980

E892AE9F-F3BD-5472-ABE8-CE924C0B28CA

Fig. 7

##### Material examined.

***Holotype***, ♂: (white, printed): 西藏普兰科加 [Xīzàng Pǔlán Kējiā] / 3700公尺 [Gōng Chǐ] / 中国科学院 [Zhōngguókēxúeyuàn] / 14. VII 1976 / 黄复生 [Huáng Fùshēng]: IOZ(E)905056 // Paratype (1♂): same data as holotype except IOZ(E)905057.

##### Redescription.

**Holotype, male.** Measurements (in mm): standard length: 5.60; pronotal length: 1.85; pronotal width: 2.10; elytral length: 3.70; elytral width: 3.00; rostral length: 1.10; rostral width: 1.00.

**Figure 7.**
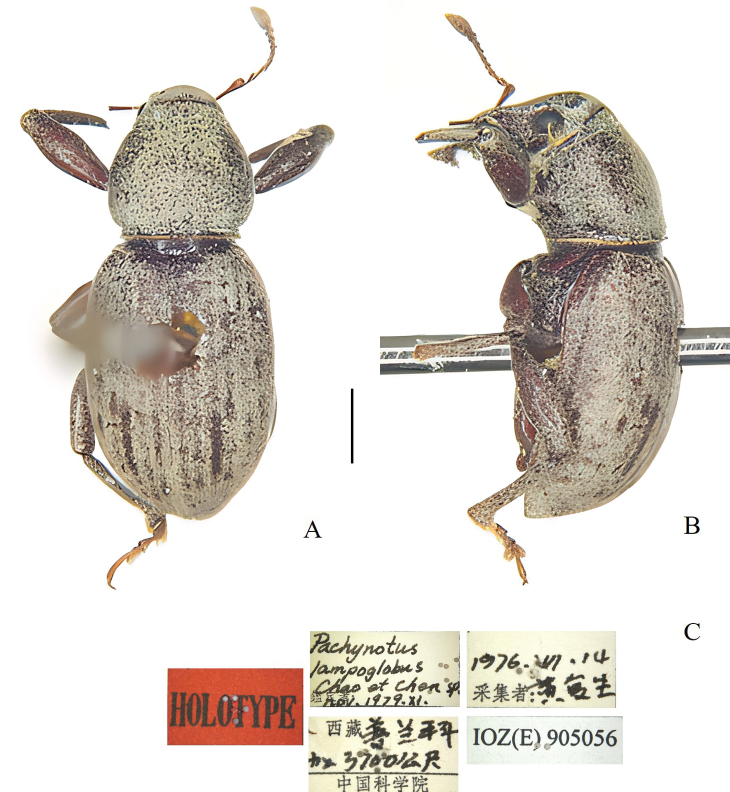
Habitus of *Pachynotuslampoglobus*, holotype. **A** dorsal view **B** lateral view **C** specimen label. Scale bars: 1 mm.

***Habitus and colour*** (Fig. 7): body wide, ovate; blackish-reddish brown; antennae and legs reddish brown, with pale yellowish-brown scales with red-green metallic lustre; scales on dorsal and lateral surfaces of rostrum moderately dense, oval to elongate-oval; antennal scape and funicles without scales; pronotum with polygonal scales, moderately dense, not contiguous; scales on elytra polygonal, dense, but not contiguous; scales on ventrites moderately dense, polygonal to elongate-oval; scales on legs dense; dorsal of tarsi surface without scales; body with erect to suberect, short, sparse setae; rostrum sparsely covered with suberect, fine, short setae; antennal scape and desmomeres I–VII with long, fine, sparse setae; dorsal and lateral surfaces of pronotum with sparse, suberect setae; setae on interstriae short, erect to suberect, equal to 2 × diameter of scale; setae on the ventral surface moderately long, fine, erect.

***Head***: convex; dorsal surface smooth; punctures small and dense; eyes moderately convex, oval; forehead convex, moderately elevated than base of rostrum in lateral view.

***Rostrum***: in dorsal view, 1.10 × as long as wide; sides narrowed from base to apex; apex narrower than base; dorsal surface with a broad and deep median sulcus, reaching middle of forehead; posterior angle of epistome more than 90°, smooth; mandibular scars oval; ventral margin of antennal scrobes visible at apical two-thirds in dorsal view; in lateral view, without triangular depression positioned laterally between eyes and antennal scrobes; prementum with four setae.

***Antennae***: scape slender, clavate, reaching middle of eyes at rest, 0.88 × length of funicle; desmomere I 1.30 × length of II, both segments elongate clavate; desmomere III short, clavate, 0.47 × length of II; desmomere IV 0.88 × length of III, nearly equal width; desmomeres IV–VI moniliform, equal in length and width; desmomere VII moniliform, 1.09 × length, 1.06 × width of VI; club elongate-oval, apical sharp, three-segmented, uniformly pubescent, segment I 1.35 × length of II; segment II shorter than segment III.

***Pronotum***: 0.88 × as long as wide; subquadrate in dorsal outline, strongly convex; sides strongly rounded, greatest width at midpoint, gradually narrowing towards both ends, fore margin shorter than posterior one; median sulcus, fine, extremely shallow; dorsal surface of pronotum smooth, punctures small, each puncture covered by a scale; postocular lobes absent, vibrissae fine, dense, yellow.

***Scutellum***: large and distinct, U-shaped, shiny, uncoated, reddish brown.

***Elytra***: 1.23 × as long as wide, moderately convex, elongate-oval; base not raised as prominent flange, not bisinuate; sides subparallel before declivity, only slightly narrowed near the base; striae distinct, narrow, moderately deep, linear; punctures minute, spot-like, moderately dense, spaces between punctures narrower than diameter of punctures; interstriae wide, flat, without tubercles, odd interstriae not more raised than even ones.

***Abdomen***: sternite I depressed in middle, slightly convex at sides; suture between sternite I and II slightly bisinuate; sternite II slightly convex; sternite I longer than II, sternite II slightly shorter than III and IV combined; sternites III and IV equal in length; sternite V moderately convex, apical round, shorter than sternites III and IV combined.

***Legs***: slightly short; femora clavate, densely with scales; fore tibiae bent inward at apical half, apex projected inwards and outwards; inner margin of fore tibiae with several moderately large teeth; median- and hind tibiae with moderately small teeth; corbels of hind tibiae closed.

##### Variation.

**Male paratype.** Measurements (in mm): standard length: 6.10; pronotal length: 1.90; pronotal width: 2.20; elytral length: 3.80; elytral width: 3.10; rostral length: 1.10; rostral width: 0.90.

**Female.** Unknown.

##### Distribution.

China (Xizang).

### ﻿Genetic distances

The intraspecific genetic distances of *P.lampoglobus* and *P.pilosus* are 0% and 1.38%, respectively (0.69%, average). The interspecific genetic distances are between 22.64% and 23.50% (23.07%, average) (Table 1).

**Table 1.** Genetic distance of *Pachynotuslampoglobus* and *P.pilosus* based on COI sequences.

	Species	GenBank Accession Number	1	2	3
1	*P.lampoglobus*	OR123593			
2		OR123594	0%		
3	*P.pilosus* sp. nov	OR123595	22.64%	22.64%	
4		OR123596	23.50%	23.50%	1.38%

### ﻿Key to species of *Pachynotus* occurring in China

**Table:** 

1	Prementum with two setae; scape reaching posterior margin of eyes at rest; inner margin of fore tibiae with extremely small teeth; mid and hind tibiae without teeth; elytral odd interstriae more raised than even ones	**2**
–	Prementum with four setae; scape reaching middle of eyes at rest; inner margin of fore tibiae with several moderately big teeth; mid and hind tibiae with moderately small teeth; elytral odd interstriae not more raised than even ones	***P.lampoglobus***
2	Scutellum triangular; rostrum with a narrow and shallow median sulcus, reaching fore margin of forehead; posterior angle of epistome > 90°; elytral base not raised as prominent flange; fore tibiae slightly bent inward at apical quarter	***P.pilosus* sp. nov.**
–	Scutellum U-shaped; rostrum with a broad and deep median sulcus, reaching head vertex; posterior angle of epistome 90°; elytral base raised as prominent flange; fore tibiae obviously bent inward at apical third	***P.arcuatus* sp. nov.**

## ﻿Discussion

The three Chinese *Pachynotus* species occurring in Xizang are all endemic to China, and morphological variation and genetic differences among these species are conspicuous. In our study, the average interspecific genetic distance between *P.lampoglobus* and *P.pilosus* was 33 × that of the average intraspecific genetic distance. This confirm, by molecular means, the validity of the new species *P.pilosus* sp. nov. (Hebert et al. 2003, 2004). In addition, the COI sequences of *P.lampoglobus* and *P.pilosus* are provided for the first time, which will help promote further DNA barcoding studies of this genus.

*Pachynotus* species in China are quite different in size from other *Pachynotus* species. *P.globicollis* (length 9.63 mm, width 2.89 mm), *P.mayarami* (length 9.55 mm, width 3.10mm), and *P.kumaonensis* (length 9.80 mm, width 3.25 mm) are larger than *P.lampoglobus* (average length 5.82 mm, average width 3.05 mm), *P.pilosus* (average length 5.12 mm, average width 2.21mm), and *P.arcuatus* (length 5.90 mm, average width 3.00 mm). It is worth noting that *P.globicollis*, *P.mayarami*, and *P.kumaonensis* are distributed in Uttarakhand, India; *P.globicollis* was found at an altitude of 1753–2286 m in Uttarakhand (Mahendiran and Ramamurthy 2013), and the other two species have no altitudinal information. *P.lampoglobus*, *P.pilosus*, and *P.arcuatus* are found in Xizang of China at altitudes of 3700 m, 4989 m, and 4685 m, respectively. This phenomenon of extreme altitude could be attributed to the advantages that diminutive bodies offer in the face of the relatively harsh conditions of high-altitude environments. In such conditions, these smaller weevils can thrive on minimal food resources and easily seek refuge by burrowing into rock crevices, thus evading predators and harsh climatic conditions.

As flightless weevils, the genus *Pachynotus* is inherently predisposed to geographical isolation, leading to the emergence of new species as observed in previous studies (Chen 1991; Ren 2008) and this study. Therefore, our findings suggest that there may be more *Pachynotus* species awaiting discovery, considering the geographic isolation and the unique ecological niches that *Pachynotus* species inhabit.

## Supplementary Material

XML Treatment for Pachynotus

XML Treatment for Pachynotus pilosus

XML Treatment for Pachynotus arcuatus

XML Treatment for Pachynotus lampoglobus

## ﻿Additional information

### Author ORCIDs

Jinliang Ren https://orcid.org/0009-0000-9847-1035

Li Ren https://orcid.org/0000-0002-8597-0449

Runzhi Zhang https://orcid.org/0000-0001-9001-0154

### Data availability

All of the data that support the findings of this study are available in the main text.

## ﻿Appendix 1

**Checklist to species of the genus *Pachynotus***

***Pachynotusglobicollis* Kollar & L. Redtenbacher, 1844: 544**

*Cneorhinusobscurus* Kollar & L. Redtenbacher, 1844: 544.

**Distribution**. India (Kashmir, Himachal Pradesh, Uttarakhand).

***Pachynotuskumaonensis* Mahendiran & Ramamurthy, 2013: 82**

**Distribution**. India (Uttarakhand).

***Pachynotuslampoglobus* Chao & Y.-Q. Chen, 1980: 88**

**Distribution**. China (Xizang).

***Pachynotusmayarami* Mahendiran & Ramamurthy, 2013: 82**

**Distribution**. India (Uttarakhand).

***Pachynotuspilosus* J.-L. Ren, Ren & Zhang, sp. nov.**

**Distribution**. China (Xizang).

***Pachynotusarcuatus* J.-L. Ren, Ren & Zhang, sp. nov.**

**Distribution**. China (Xizang).
